# Kyphosis and Sleep Characteristics in Older Persons: The Rancho Bernardo Study

**DOI:** 10.23937/2572-4053.1510004

**Published:** 2015-09-21

**Authors:** Che Wankie, D. Kritz-Silverstein, E. Barrett-Connor, D.M. Kado

**Affiliations:** 1 Joint Doctoral Program in Public Health (Epidemiology), San Diego State University/University of California, USA; 2 Department of Family Medicine and Public Health, University of California, USA

**Keywords:** Kyphosis, Flexicurve, Sleep quality, Older persons

## Abstract

**Background:**

Kyphosis is a forward curvature of the thoracic spine that is associated with multiple adverse health outcomes. This cross-sectional study examined the association between kyphosis and sleep characteristics.

**Methods:**

Participants were 468 white, community-dwelling individuals (women = 255; men = 213) from the Rancho Bernardo cohort who had kyphosis assessed using a flexicurve ruler at a 2007–09 follow-up research clinic visit and sleep quality assessed by mailed survey in 2010 with the Pittsburgh Sleep Quality Index (PSQI), scored 0–18, with >5 indicative of poor sleep quality.

**Results:**

Women had a mean age of 73.3 ± 8.8 years; men 74.2 ± 8.1 years. Mean flexicurve measures were 12.6 ± 3.2 for women and 12.1 ± 2.6 for men. No significant associations were found between kyphosis and any self-reported sleep measure in men, but women with worse kyphosis had poorer sleep quality, based on total PSQI score and two PSQI subcomponents. In women, with each unit increase in kyphosis, after adjusting for age, marital status, height, general health, calcium supplement use, estrogen use, exercise, arthritis, and depression, there was an associated increase in total PSQI score, indicating worse sleep quality (standard β-estimate = 1.37, 95% CI: 1.03, 1.82). Women with worse kyphosis were also more likely to sleep ≤ 7 hours (Odds Ratio (OR) = 1.11, 95% CI: 1.02, 1.22) and report use of sleep medications (OR = 1.14, 95% CI: 1.03, 1.25).

**Conclusions:**

In women only, those with worse flexicurve kyphosis had worse scores on the PSQI, slept fewer hours (≤ 7 hours) and were more likely to report sleep medication use than those with less kyphosis. The association between kyphosis and objective sleep measures in older persons deserves further investigation.

## Introduction

Accentuated kyphosis, popularly known as the dowager’s hump, is a forward curvature of the thoracic spine that appears as a humped or crooked back [1,2]. Progressive kyphosis may develop as a result of spinal fractures caused by osteoporosis, postural changes due to muscle weakness, and/or degenerative disc disease [3,4]. Hyperkyphosis is a common condition affecting 20–40% of older persons, but can occur less commonly among the young [2]. In older persons, hyperkyphosis has been associated with falls, fractures, disability, and mortality [5–9]. Thoracic kyphosis is also associated with mild back pain and restricted respiratory function [4,10]. Given these previous observations, it is conceivable that hyperkyphosis associated with physical discomforts including impaired respiratory function may contribute to the deterioration of sleep quality.

Sleep is an important physiological need for human health. Sleep complaints are frequent among older persons [11,12]; the National Sleep Foundation reported that in 2003 about 67% of adults aged 65 years and older had at least one sleep-related complaint [13]. Moreover, sleep disorders are often unrecognized and untreated among older persons [14]. Although sleep changes may occur with age alone, it is plausible that the sleep-wake schedule of persons with kyphosis may be further altered due to weak spinal extensor muscles, disturbed breathing patterns, and pressure exerted on the diaphragm which may make sleep an uncomfortable experience. However, to our knowledge, no previous study has examined the association of kyphosis with sleep quality.

To investigate whether hyperkyphosis is associated with sleep quality in older persons, we used data from a large community-dwelling sample of older adults. We hypothesized that older men and women with hyperkyphosis would report worse sleep habits based upon focus group reports of worse posture leading to worse self-reported sleep [15]. If confirmed, complications from poor sleep might serve as an explanation for some of the previously described associations of hyperkyphosis with poor physical functioning, falls, fractures, and earlier mortality.

## Methods

### Participants

Participants were residents of Rancho Bernardo, a southern California community, who were enrolled in a population-based study of heart disease risk factors first established in 1972–1974. This cohort consists of predominantly white, middle-class, community-dwelling individuals ranging in age from 49 to 97 years, who since enrollment, participated in periodic research clinic visits and completed yearly mailed surveys. In 2007–2009, surviving members of the cohort aged 50 years and older who had attended one or more of the previous clinic visits were invited to attend a follow-up research clinic visit to obtain repeat measures of bone mineral density and kyphosis. In 2010, all surviving members of the original cohort were mailed survey collecting information about sleep quality.

Of the 733 participants who attended the 2007–2009 follow-up research clinic visit and had kyphosis measured, 580 participants also completed the mailed questionnaire that included 17 items from the Pittsburgh Sleep Quality Index (PSQI) questionnaire. After excluding 112 participants who did not complete all PSQI questionnaire items, there remained 468 (women = 255, men = 213) included in this study sample. This study was approved by the University of California, San Diego Human Subjects Protections Program. All participants gave written, informed consent prior to participating.

### Procedures

At the 2007–09 follow-up clinic visits, self-reported information on demographic characteristics (gender, age, and marital status) was collected. In the clinic, height and weight were measured with participants in light clothing and without shoes, enabling calculation of body mass index (BMI = weight [kg]/height [m^2^]) as an estimate of obesity. Self-administered surveys were used to collect information on smoking status (never, past, and current), frequency of alcohol use (daily or almost daily, 3–4 times a week, 1–2 times a week, 1–2 times a month, and less often than once a month), and exercise three or more times a week (yes/no). Participants were also queried about their medical history, including doctor’s diagnosis of diabetes, arthritis, and heart disease. Medication use, including calcium supplement use, vitamin D use, and estrogen use (in women) was queried and validated with pills and prescription containers brought to the clinic for that purpose.

## Kyphotic Posture Measurement

**Figure 1:**
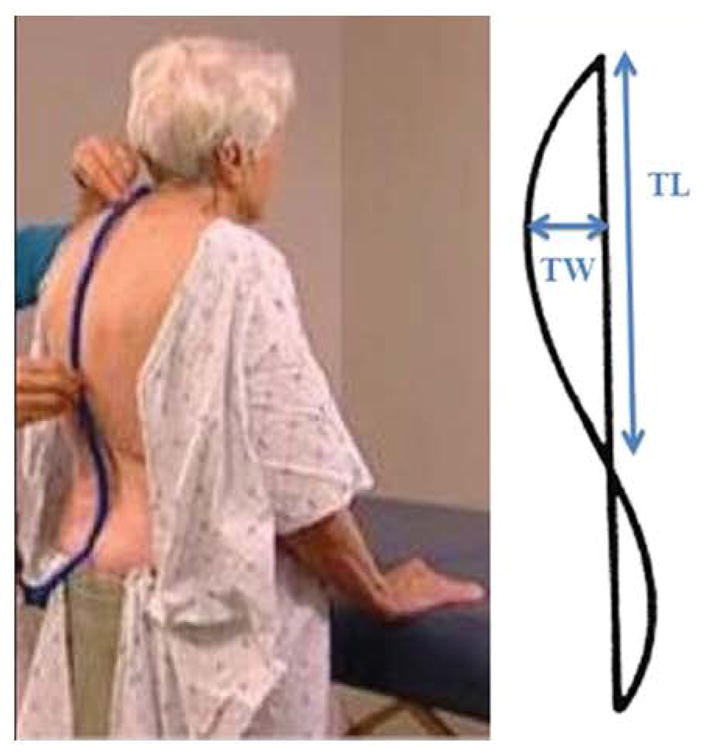
Flexicurve measure of kyphotic posture Flexicurve ruler and traced contour on graph paper TL: Thoracic length TW: Thoracic width Source: Reprinted Katzman et al., (2010).

Kyphosis was assessed using the flexicurve ruler (Figure 1) (Staedtler Mars Inc. Numberg, Germany). The ruler is a strip of flexicurve metal covered in plastic and approximately 60 cm in length. The ruler was placed on the back of the participant standing in his/her usual best position, with the top end starting at C7, and gently bent so that it conformed to the contour of the participant’s backbone. The shape of the flexicurve ruler was then traced onto graph paper and a straight line was drawn connecting both ends of the curve. The thoracic height (also referred to as thoricic width) was determined as the distance between the straight line and the vertex of the curve. The thoracic length was determined as a measure of the straight line that formed the base of the convex posteriorly. A flexicurve kyphosis index was computed as a ratio of the height and length measurements multiplied by 100: (height/length) x 100.

## Sleep Quality Assessment

Information about sleep was obtained from a 2010 mailed survey that included 17 of 19 questions from the Pittsburgh Sleep Quality index (PSQI). The PSQI items are collapsed into components through a complex algorithm to generate a global score that reflects the sleep quality of the participant [16]. For this study, the PSQI global score was computed using six of the seven components. The components used were: (1) subjective (self-reported) sleep quality-- indicated by how rested an individual feels after their sleep; (2) sleep onset latency-- the length of time between going to bed and falling asleep; (3) sleep duration-- the total length of time one spends asleep during the whole night; (4) sleep efficiency-- is the proportion of time that one is asleep relative to the total time spent in bed; (5) sleep disturbances-- conditions or factors that affect an individual’s sleep such as pain, feeling too hot or cold, and breathing discomfort; and (6) use of sleeping medication-- the use of sleeping pills obtained either over-the-counter or prescribed by a physician. The PSQI global score used in this study ranged from 0–18; lower scores indicate better sleep quality. An individual was classified as having good sleep quality if they had a global score of 5 or less [17].

## Statistical Analysis

Internal consistency of the total PSQI was assessed with Cronbach’s alpha. Sleep outcome measures were analyzed as both continuous and categorical variables. Descriptive statistics were used to summarize the main outcome (sleep quality), the main exposure (flexicurve kyphotic measure), and all potential covariates. Characteristics were summarized as means ± SD for continuous variables and as counts and percentages for categorical variables. Skewed variables were log-transformed for multivariate analysis and back-transformed for interpretation.

After a comprehensive literature review we assembled a list of potential confounders of the association between kyphosis and sleep quality. We used Independent t-tests, the chi-square test of homogeneity and the Fisher’s exact test to assess significant associations between each candidate variable and sleep quality. A variable was considered a potential confounder and added into the full multivariate model if it met the significance criteria of p < 0.10.

The PSQI global index score was used as the primary outcome while the six components used to compute this index were each used as separate secondary outcomes. Cut-off criteria were based on PSQI clinimetric properties and several prior sleep studies that validated the PSQI psychometric properties [16–20]. Sleep variables used for analysis were categorized as follows: PSQI score (poor sleep quality if PSQI > 5 versus good sleep quality PSQI ≤ 5), sleep duration (≤ 7 hours versus > 7 hours), sleep efficiency (poor efficiency if ≤ 85% versus good efficiency if > 85%), sleep onset latency (good latency if ≤ 30 minutes versus poor if > 30 minutes), self-reported sleep quality (good versus poor), sleep disturbance (frequent versus less frequent), and use of sleep medication (frequent versus less frequent). Sleep disturbance and use of sleep medication were defined as a frequent event if it occurred more than once a week [16,21]. Sleep duration was examined for any U-shaped associations with kyphosis measures. Kyphosis was examined as a unit increase in flexicurve measure because there are no established guidelines to define flexicure hyperkyphosis.

Unadjusted and age-adjusted, sex-specific comparisons of flexicurve measures by sleep quality (good vs. poor) were performed with independent t-tests and analysis of covariance (ANCOVA). Linear regression was used to examine the relation between kyphosis and sleep outcomes analyzed as continuous variables. Three models were examined for each sleep quality outcome: unadjusted, age-adjusted, and the full model that included all candidate variables that met the screening criteria of being associated with sleep quality (age, marital status, height, general health, calcium supplement use, exercise, arthritis, depression, and estrogen use). Results from linear regression analyses were presented as standardized beta estimates after adjusting for covariates. Multiple logistic regression was used to examine the association between kyphosis and each dichotomized sleep outcome (sleep quality, sleep efficiency, sleep onset latency, self-reported sleep quality, sleep disturbance, and use of sleep medication) after adjusting for covariates. Odds ratios and 95% confidence intervals were presented for logistic regression models after adjusting for covariates.

Sensitivity analyses assessed the association between kyphosis and sleep quality using a PSQI cutoff of 4.3 instead of 5 to proportionately adjust for use of only six of the seven available components used to compute the PSQI global index score. All statistical tests were 2-tailed with p < 0.05 considered statistically significant. Data were analyzed using SAS version 9.3 for Windows (SAS Institute, Inc., Cary, North Carolina).

## Results

### Sample characteristics

The six component scores of the PSQI had an overall reliability Cronbach’s alpha coefficient of 0.72, indicating the scale was reliable with the sample used. There were some significant sex differences in study baseline characteristics (see Table 1). At baseline in 2007–2009, more participants were women and the mean age of participants was 73.7 years (SD = 8.5 years). On average, compared to men, women were significantly shorter, had a lower weight and body mass index, were less likely to have been married, were more kyphotic, and more likely to report poor sleep quality. They also were more likely to report using calcium and vitamin D supplements, and had a higher prevalence of arthritis and depression.

Table 2 shows gender-specific comparisons of characteristics according to self-reported sleep quality. In women only, those who reported poor sleep quality had higher mean weight and body mass index, reported poorer health, were more likely to be a past-smoker, less likely to exercise three or more times per week, and had a higher prevalence of arthritis and depression. Among men, compared to those who reported good sleep quality, those with poor sleep quality had a higher prevalence of arthritis.

### Associations of kyphosis with sleep quality

The associations of kyphosis with sleep quality based on PSQI scores as a continuous and as a dichotomized variable (> 5) are shown in Table 3. Among women only, age- and multivariate adjusted analyses showed that for every standard deviation increase in the flexicurve measure, the PSQI global score (as a continuous variable) significantly increased by 1.4 standard deviation units, indicating that women with more kyphosis had poorer sleep quality. The association between kyphosis measure and sleep quality based on the dichotomized PSQI global score (poor sleep quality > 5) was not statistically significant both before and after adjustment for covariates. Among men, there were no significant associations of kyphosis with PSQI scores of sleep quality.

Table 4 shows the association of kyphosis with each component of the PSQI questionnaire. In women, there was a significant association between kyphosis and sleep duration after adjusting for age and other covariates. Each standard deviation increase in the flexicurve measure was associated with a 0.18 unit decline in sleep duration, indicating that women with more kyphosis slept fewer hours than those with less kyphosis. Likewise, for every unit increase in flexicurve kyphosis, the age-adjusted odds of sleeping 7 hours or less (relative to more than 7 hours) increased by 11% in the age-adjusted model (95% CI = 1.02, 1.20) and by 13% in the multivariate adjusted model (95% CI = 1.03, 1.23). Additionally, among women only, the odds of reporting frequent use of sleep medication (relative to infrequent use of sleep medication) increased by 11% in age-adjusted (95% CI = 1.01, 1.22) and by 14% in multivariate adjusted models (95% CI = 1.03, 1.25) for every unit increase in flexicurve measure. There were no significant associations between kyphosis and sleep measures in men in any of the models, both before and after adjustment for covariates.

## Discussion

Results from this study suggest that there is an association between greater kyphosis curvature and self-reported poor sleep quality among older women, but not older men. In the overall analysis that defined poor sleep quality by increasing PSQI score, each standard deviation increase in flexicurve index was associated with worse self-reported sleep quality in women. Furthermore, when examining specific components of the PSQI, women with worse kyphosis were more likely to sleep 7 hours or less and to report frequent use of sleep medications. These associations remained significant even after further adjustment for age, marital status, height, general health, calcium supplement use, exercise, arthritis, depression, and estrogen use. Among men, there were no significant associations between kyphosis and any of the measures of sleep quality, before and after adjusting for age and other covariates.

Although osteoporotic-related vertebral fractures have been associated with poor sleep and are well recognized as a consequence of kyphosis, there is a dearth of information about the association of kyphosis with sleep quality [22]. To our knowledge, apart from a focus-group discussion involving patients with osteoporosis and kyphosis [15], no previous study has examined the association of kyphosis and sleep quality in older persons. It is plausible that greater degrees of kyphosis cause chest wall restriction and impaired muscle mobility, ultimately resulting in impaired pulmonary function that could in turn, affect sleep quality [23].

It is unclear why sleep quality decreases with increasing flexicurve scores among women but not men. A previous study that examined the health status of adolescents with common spinal deformities versus individuals without deformity using the Quality of Life Profile for Spine Deformities (QLPSD) showed that girls with spinal deformities were on average more than nine times (p = 0.028) more likely to have problems with sleeplessness than boys [24]. Alternatively, it is possible that, for women, increased flexicurve measures resulting from vertebral fractures may explain the decrease in sleep quality. Vertebral fractures, which have been often cited as the cause of kyphosis, are more common among women compared with men [2]. Finally, it is also possible that women might be more likely than men to self-report problems related to sleep.

It should be noted that our study did not find that women or men with worse kyphosis had poor sleep as standardly defined by the PSQI cut-off of 5 or more. However, we used a modified version of the PSQI that included only six of the seven original components so perhaps the cut-off of 5 or more to define sleep might have been too high a threshold given that there were fewer items on the questionnaire to score. However, using a proportionally adjusted cutoff of 4.3 to define poor sleep did not change the results. Although women with worse kyphosis did not seem more likely to have overall poor sleep as defined by a validated questionnaire, our study findings do suggest an important association between worse kyphosis and specific measures of unhealthy sleep behaviors that include less sleep duration and frequent sleep medication use. If the association between hyperkyphosis and poor sleep characteristics is confirmed in other studies, clinical interventions may be considered such as screening for poor sleep in those with hyperkyphosis. In addition, developing treatment strategies that focus on posture may not only help prevent age-related kyphosis progression, but could also potentially improve sleep. For those who do suffer from insomnia, having alternative new treatments is desirable. While the currently available pharmacologic sleep aids are helpful, the use of sedative hypnotics is associated with falls and cognitive impairment [25,26].

Several limitations of this study should be noted. This study was cross-sectional and therefore could not determine causality between kyphosis and sleep quality. Another limitation is that unfortunately, one component of the PSQI was inadvertently omitted, thus the total score used for this study was based on only 6 of the 7 components of the validated PSQI questionnaire. The missing item was information about participants’ daytime dysfunction due to sleepiness. Using the recommended PSQI cut-off (greater than 5), could have led to an underestimation of levels of poor sleep quality but using a lower threshold of 4.3 yielded similar results. It should also be noted that sleep measures were obtained as subjective reports and that women may be more likely than men to report sleep-related problems. Objective measures of sleep were not available in this study to confirm the subjective reports of sleep quality. Finally, findings from this study may not be generalizable to other populations because the participants were predominantly well-educated, white, middle class, community-dwelling adults.

There are also several strengths to this study. Although the sample for this study consisted of predominantly white, well-educated, middle to upper-middle class participants that may compromise generalizability, confounding due to socioeconomic status and lack of access to medical care is limited. This study uses data from a large population based community-dwelling cohort rather than a clinic-based cohort that may have higher rates of sleep problems. This study also included both older men and women, whereas many kyphosis studies to date included only women.

In conclusion, our results provide preliminary confirmation that worse degrees of kyphosis may be associated with sleep problems in older women. Additional research using objective sleep measures to explore the association between kyphosis and sleep quality is needed.

## Figures and Tables

**Table 1:** Sample characteristics by sex; Rancho Bernardo, California

Characteristics	Total sample (n = 468) n (%)	Women (n = 255) n (%)	Men (n = 213) n (%)	*p*-value
Age, [*years*], mean ± *SD*	73.7 ± 8.5	73.3 ± 8.8	74.2 ± 8.1	0.28
Height, [*in.*], mean ± *SD*	65.9 ± 3.8	63.3 ± 2.5	69.0 ± 2.8	< 0.01
Weight, [*lb.*], mean ± *SD*	165.8 ± 36.5	147.8 ± 30.2	187.2 ± 31.6	< 0.01
BMI, [*lb./in^2^* ], mean ± *SD*	26.7 ± 4.6	25.9 ± 4.9	27.7 ± 4.1	< 0.01
Marital Status (n = 468)				< 0.01
Married/Living together as if married	364 (77.8)	172 (67.5)	192 (90.1)	
Widowed/Divorced/Separated	96 (20.5)	77 (30.2)	19 (8.9)	
Single	8 (1.7)	6 (2.4)	2 (0.9)	
Kyphosis				
Flexicurve, mean ± *SD*	12.4 ± 3.0	12.6 ± 3.2	12.1 ± 2.6	0.04
PSQI				< 0.01
Good sleep quality	303 (64.7)	151 (59.2)	152 (71.4)	
Poor sleep quality	165 (35.3)	104 (40.8)	61 (28.6)	
General health (n = 468)				0.87
Excellent/Very good	297 (63.5)	161 (63.1)	136 (63.9)	
Good/Fair/Poor	171 (36.5)	94 (36.9)	77 (36.1)	
Smoking status (n = 467)				0.13
Never	239 (51.2)	140 (55.1)	99 (46.5)	
Past	211 (45.2)	104 (40.9)	107 (50.2)	
Current	17 (3.6)	10 (4.0)	7 (3.3)	
Alcohol (n = 410)				0.16
Daily or almost every day	171 (41.7)	83 (48.1)	88 (45.8)	
Three or four times a week	58 (14.2)	29 (13.3)	29 (15.1)	
Once or twice a week	58 (14.2)	34 (15.6)	24 (12.5)	
Once or twice a month	47 (11.4)	23 (10.5)	24 (12.5)	
Less often than once a month	76 (18.5)	49 (22.5)	27 (14.1)	
Calcium supplement use, % Yes (n = 468)	210 (44.8)	144 (56.5)	66 (31.0)	< 0.01
Vitamin D current, % Yes (n = 468)	197 (42.1)	114 (56.5)	53 (24.9)	< 0.01
Estrogen ever, % Yes (n = 251)	195 (77.7)	195 (77.7)	-	-
Estrogen current, % Yes (n = 195)	45 (23.1)	45 (23.1)	-	-
Exercise ≥ three times a week, %Yes (n = 467)	336 (72.0)	174 (68.5)	162 (76.1)	0.07
Emphysema, % Yes (n = 457)	14 (3.1)	11 (4.4)	3 (1.5)	0.07
Diabetes, % Yes (n = 468)	51 (10.9)	21 (8.2)	30 (14.1)	0.04
Arthritis, % Yes (n = 476)	96 (20.6)	63 (24.9)	33 (15.5)	0.01
Depression, % Yes (n = 467)	57 (12.2)	38 (14.9)	19 (9.0)	0.05

Abbreviations: SD = standard deviation; *in.* = inches; *lb.* = pound; PSQI = Pittsburgh Sleep Quality Index; Good sleep quality (PSQI ≤ 5)

**Table 2:** Sample characteristics by sex and PSQI sleep quality; Rancho Bernardo, California

	Women			Men		
Characteristics	Good Sleep Quality (n = 151)	Poor Sleep Quality (n = 152)	*p*-value	Good Sleep Quality (n = 104)	Poor Sleep Quality (n = 64)	*p*-value
Age, [*years*], mean ± *SD*	72.5 ± 9.1	74.6 ± 8.4	0.06	74.2 ± 7.9	74.1 ± 8.7	0.94
Height, [*in*], mean ± *SD*	63.3 ± 2.4	63.3 ± 2.6	0.91	68.8 ± 2.7	69.4 ± 2.9	0.20
Weight, [*lb.*], mean ± *SD*	144.2 ± 28.3	153.1 ± 32.1	0.02	186.1 ± 32.9	190.1 ± 27.9	0.37
BMI, [*lb./in^2^* ], mean ± *SD*	25.3 ± 4.5	26.9 ± 5.4	0.01	27.6 ± 4.2	27.9 ± 3.9	0.71
Marital Status (n = 468)			0.22			1.00
Married/Living together as if married	108 (71.5)	64 (61.5)		136 (89.5)	56 (91.8)	
Widowed/Divorced/Separated	40 (26.5)	37 (35.6)		14 (9.2)	5 (8.2)	
Single	3 (2.0)	3 (2.9)		2 (1.3)	0 (0.0)	
Kyphosis						
Flexicurve, mean ± *SD*	12.4 ± 3.3	12.9 ± 3.0	0.25	12.2 ± 2.6	11.9 ± 2.6	0.49
General health (n = 468)			0.02			0.01
Excellent/Very good	104 (68.7)	57 (54.8)		105 (69.1)	31 (50.8)	
Good/Fair/Poor	47 (31.1)	47 (45.2)		47 (30.9)	30 (49.2)	
Smoking status (n = 467)			0.03			0.06
Never	88 (58.3)	52 (50.5)		77 (50.6)	22 (36.1)	
Past	54 (35.8)	50 (48.5)		72 (47.4)	35 (57.4)	
Current	9 (6.0)	1 (1.0)		3 (2.0)	4 (6.5)	
Alcohol (n = 410)			0.73			0.30
Daily or almost every day	46 (35.9)	37 (41.2)		67 (49.3)	21 (37.5)	
Three or four times a week	18 (14.1)	11 (12.2)		20 (14.7)	9 (16.1)	
Once or twice a week	23 (18.0)	11 (12.2)		18 (13.2)	6 (10.7)	
Once or twice a month	12 (9.4)	11 (12.2)		13 (9.6)	11 (19.6)	
Less often than once a month	29 (22.6)	20 (22.2)		18 (13.2)	9 (16.1)	
Calcium supplement use, % Yes (n = 468)	81 (53.6)	63 (60.6)	0.27	43 (28.3)	23 (37.7)	0.18
Vitamin D current, % Yes (n = 468)	81 (53.6)	63 (60.6)	0.27	33 (21.7)	20 (32.8)	0.09
Estrogen ever, % Yes (n = 251)	116 (77.3)	79 (78.2)	0.87	-	-	
Estrogen current, % Yes (n = 195)	26 (22.4)	19 (24.1)	0.79	-	-	
Exercise ≥ 3 times/week, %Yes (n = 467)	110 (73.3)	64 (61.5)	0.05	114 (75.0)	48 (78.7)	0.57
Emphysema, %Yes (n = 457)	5 (3.4)	6 (5.8)	0.37	1 (0.7)	2 (3.4)	0.19
Diabetes, %Yes (n = 468)	9 (6.0)	12 (11.5)	0.11	22 (14.5)	8 (13.1)	0.80
Arthritis, %Yes (n = 476)	24 (16.0)	39 (37.9)	<0.01	17 (11.2)	16 (26.2)	<0.01
Depression, %Yes (n = 467)	17 (11.3)	21 (20.2)	0.05	10 (6.6)	9 (14.7)	0.06

Abbreviations: SD = standard deviation; *in.* = inches; *lb.* = pound; PSQI = Pittsburgh Sleep Quality Index

**Table 3:** Unadjusted, age- and multivariate adjusted associations of kyphosis with PSQI sleep quality in women and men; results of linear and logistic regression analysis

	Flexicurve	
	Women (n = 255)	Men (n = 213)
Sleep measures	β^b^ (95% CI)	β^b^ (95% CI)
PSQI *(range 0–18)*		
Unadjusted	**1.40 (1.05, 1.85)**	0.93 (0.68, 1.28)
Age-adjusted	**1.37 (1.03, 1.82)**	0.93 (0.68, 1.28)
Multivariate adjusted a	**1.37 (1.03, 1.82)**	0.87 (0.64, 1.19)
	**OR (95% CI)**	**OR (95% CI)**
PQSI *(poor sleep > 5)*		
Unadjusted	1.05 (0.97, 1.13)	0.96 (0.85, 1.08)
Age-adjusted	1.04 (0.96, 1.12)	0.96 (0.85, 1.08)
Multivariate adjusted a	1.04 (0.95, 1.13)	0.93 (0.82, 1.06)

Abbreviations: PSQI = Pittsburgh Sleep Quality Index; Good sleep quality (PSQI ≤ 5) and Poor sleep quality (PSQI > 5) are based on the PSQI = Pittsburgh Sleep Quality Index (range 0–18)

§ *p* <0.05

a Multivariate adjusted: age, marital status, height, general health, calcium supplement use, exercise, arthritis, depression and estrogen (for women)

b Standardized β-coefficient

**Table 4:** Unadjusted, age- and multivariate adjusted associations of kyphosis measures with PSQI sleep components by sex; results of linear and logistic regression analysis

	Flexicurve	
	Women (n = 255)	Men (n = 213)
Sleep measures	β^b^ (95% CI)	β^b^ (95% CI)
Sleep duration *(hours)*		
Unadjusted	**−0.16 (−0.28, −0.04)**	0.11 (−0.02, 0.25)
Age-adjusted	**−0.18 (−0.31, −0.06)**	0.09 (−0.04, 0.23)
Multivariate adjusted a	**−0.18 (−0.31, −0.06)**	0.09 (−0.04, 0.24)
Sleep efficiency *(%)*		
Unadjusted	0.91 (0.69, 1.21)	1.17 (0.86, 1.60)
Age-adjusted	0.98 (0.73, 1.30)	1.19 (0.87, 1.63)
Multivariate adjusted a	0.92 (0.69, 1.24)	1.19 (0.86, 1.65)
Sleep onset latency *(minutes)*		
Unadjusted	1.33 (1.00, 1.77)	−0.04 (−0.02, 0.01)
Age-adjusted	1.25 (0.94, 1.66)	−0.05 (−0.02, 0.01)
Multivariate adjusted a	1.26 (0.95, 1.68)	−0.06 (−0.03, 0.01)
	**OR (95% CI)**	**OR (95% CI)**
Sleep duration (*≤ 7 hours)* c		
Unadjusted	**1.10 (1.02, 1.19)**	0.93 (0.84, 1.04)
Age-adjusted	**1.11 (1.02, 1.20)**	0.94 (0.85, 1.05)
Multivariate adjusted a	**1.11 (1.02, 1.22)**	0.94 (0.84, 1.06)
Sleep efficiency (*≤ 85%)* c		
Unadjusted	0.95 (0.87, 1.03)	1.04 (0.93, 1.17)
Age-adjusted	0.97 (0.89, 1.05)	1.05 (0.94, 1.18)
Multivariate adjusted a	0.96 (0.88, 1.05)	1.06 (0.94, 1.19)
Sleep onset latency (*> 30 minutes)* c		
Unadjusted	1.08 (0.96, 1.20)	0.94 (0.79, 1.12)
Age-adjusted	1.04 (0.93, 1.17)	0.94 (0.79, 1.12)
Multivariate adjusted a	1.06 (0.93, 1.20)	0.89 (0.74, 1.08)
Self-reported sleep quality (*poor*) c		
Unadjusted	1.11 (0.98, 1.25)	0.86 (0.70, 1.05)
Age-adjusted	1.11 (0.98, 1.26)	0.86 (0.69, 1.05)
Multivariate adjusted a	1.09 (0.95, 1.24)	0.87 (0.70, 1.07)
Sleep disturbance (*frequent*) c		
Unadjusted	0.97 (0.89, 1.06)	1.04 (0.90, 1.21)
Age-adjusted	0.98 (0.89, 1.07)	1.04 (0.90, 1.21)
Multivariate adjusted a	0.95 (0.86, 1.05)	1.03 (0.88, 1.21)
Sleep medications (*frequent*) c		
Unadjusted	**1.12 (1.03, 1.23)**	0.96 (0.85, 1.09)
Age-adjusted	**1.11 (1.01, 1.22)**	0.96 (0.85, 1.09)
Multivariate adjusted a	**1.14 (1.03, 1.25)**	0.95 (0.82, 1.09)

Abbreviations: PSQI = Pittsburgh Sleep Quality Index; Good sleep quality (PSQI ≤ 5) and Poor sleep quality (PSQI > 5) are based on the PSQI = Pittsburgh Sleep Quality Index (range 0–18)

§ *p* <0.05

a Multivariate adjusted: age, marital status, height, general health, calcium supplement use, exercise, arthritis, depression and estrogen (for women)

b Standardized β-coefficient

c Recommended sleep duration (> 7 hours); good sleep efficiency (> 85%); good sleep onset latency (≤ 30 minutes);

Frequent sleep disturbance (more than once a week); frequent sleep medication use (more than once a week)
